# ERRATUM

**DOI:** 10.1590/1980-549720200009erratum.1

**Published:** 2023-05-08

In the manuscript “Tuberculosis and diabetes: association with sociodemographic characteristics and diagnosis and treatment of tuberculosis. Brazil, 2007-2011”, DOI: https://doi.org/10.1590/1980-549720200009, published in the Rev Bras Epidemiol. 2020; 23: E200009:

**Where it reads:**

Lúcia Santana Rolim

**It should read:**

Lúcia Rolim Santana de Freitas

